# Significance of borderline HbA_2_ levels in β thalassemia carrier screening

**DOI:** 10.1038/s41598-022-09250-5

**Published:** 2022-03-30

**Authors:** Stacy Colaco, Roshan Colah, Anita Nadkarni

**Affiliations:** Department of Hematogenetics, National Institute of Immunohematology, Indian Council of Medical Research [ICMR], 13th Floor, K.E.M. Hospital Campus, Parel, Mumbai, 400 012 India

**Keywords:** Genetics, Molecular biology, Diseases, Health care, Medical research, Molecular medicine

## Abstract

Increased HbA_2_ levels are the characteristic feature of β-thalassemia carriers. A subset of carriers however do not show HbA_2_ levels in the typical carrier range (≥ 4.0%) but show borderline HbA_2_ levels. As a result, these carriers escape diagnosis and carry the risk of having β-thalassemia major offspring. Borderline HbA_2_ values may occur as a consequence of mild β-thalassemia mutations, co-inherited β-thalassemia and α- or δ- thalassemia or iron deficiency anemia. However, there is insufficient knowledge regarding the cause of borderline HbA_2_ levels in specific populations. This study aimed to identify the determinants of borderline HbA_2_ levels (which we have considered as HbA_2_ 3.0–3.9%) in 205 individuals. Primary screening involved detecting the presence of iron deficiency anemia followed by molecular analysis of α, β and δ globin genes. Remarkably, 168 of 205 individuals were positive for a defect. 87% (149/168) of positive individuals were heterozygous for β thalassemia with (59/149) or without (90/149) the presence of co-existing IDA, α or δ gene defects. Notably, 20 of 149 β thalassemia carriers showed HbA_2_ < 3.5% and MCV > 80fL. 7 of these 20 carriers were married to carriers of hemoglobinopathies. Our findings describe the genetic basis of borderline HbA_2_ levels and emphasize the necessity of a molecular diagnosis in these individuals in the routine clinical setting.

## Introduction

The presence of a single β-thalassemia allele is usually associated with hypochromic microcytic red cells and an increase in HbA_2_ levels. In some cases, the effect of this allele or its interaction with other molecular or acquired defects may render it completely silent resulting in normal or borderline HbA_2_ levels^1^. The term borderline HbA_2_ refers to values between the upper limit of the normal range (2.0–3.2%) and the lower limit of typical β-thalassemia carriers (3.3–3.9%)^2^. Carriers showing borderline HbA_2_ levels may be missed during routine screening programs for β-thalassemia and may only be detected following the birth of affected offspring. Hence, these genotypes must be considered at-risk for having children with the disease if their partner is also a β-thalassemia carrier^3^.

Borderline HbA_2_ levels are not uncommon in populations with a high frequency of β-thalassemia such as in Italy^1,4,5^ and in Greece^6^. Individuals with borderline HbA_2_ levels have also been documented in the Middle Eastern populations^7,8^ and Pakistan^9^. A recent review has shown that individuals with borderline HbA_2_ levels have been reported in as many as 31 different countries worldwide and has suggested that migration, inter-country adoptions and inter-racial marriages may have contributed in the spread of globin gene defects in geographic locations they were not identified earlier^10^. A study from India previously reported of 18 individuals with a normal β genotype and 7 individuals heterozygous for a β thalassemia allele, all showing HbA_2_ levels between 3.0 and 4.0%^11^. Another study focussing on the North-Indian population identified a β globin gene defect in 32% of 25 individuals with HbA_2_ levels between 3.5 and 3.9%^12^. A subsequent study that included individuals referred for screening from different regions in India reported 131 β-thalassemia heterozygotes having normal or borderline HbA_2_ levels [2.4–3.5%]^13^. Based on a nation-wide report, the overall prevalence of β-thalassemia heterozygosity in India is 2.78% with variations from 1.48–3.64% in different states^14^. The current landscape of β-thalassemia in India warrants adequate importance being assigned to the detection of subjects with borderline HbA_2_ levels in the population. Presently, there is paucity of published data on understanding the heterogeneity of borderline HbA_2_ levels in Indians and knowledge of the different determinants affecting HbA_2_ levels remains largely unknown. Hence, this study was designed to evaluate the prevalence and the significance of borderline HbA_2_ values for β-thalassemia carrier screening.

## Materials and methods

### Selection of patients

The study was approved by the Institutional Ethics Committee of ICMR-NIIH, Mumbai, India. From the 16,590 individuals referred to our institute for screening of hemoglobinopathies from 2009 to 2013 we identified 370 individuals showing HbA_2_ levels between 3.0 and 3.9%. Of these 370 individuals, 205 individuals [90 males and 115 females], with age ranging between 2 and 66 years, were randomly selected for our study after obtaining written informed consent. In the case of minors parental consent was obtained. The HbA_2_ cut-off for diagnosing heterozygous β-thalassemia in our laboratory at the time this study was performed was HbA_2_ ≥ 4.0% and hence these 205 subjects were classified as “borderline HbA_2_ subjects” [3.0–3.9%].

### Primary screening and molecular analysis

10 ml of venous blood was collected from each individual in EDTA and plain vacutainers for hematological, molecular and serological analysis after verbal and written informed consent. Red cell indices were measured on an automated blood cell counter [Sysmex K-500i; Sysmex, Kobe, Japan]. Iron deficiency anemia was ruled out by measuring serum ferritin [Ferritin ELISA Kit, Demeditec Diagnostics, GmbH] and soluble transferrin receptor [Quantikine IVD Soluble Transferrin Receptor ELISA, RnD Diagnostics, R&D Systems, Minneapolis, MN, USA] levels using enzyme-linked-immunosorbent serologic assays. HbA_2_ and HbF levels were measured using cation exchange high performance liquid chromatography [HPLC] on the VARIANT Hemoglobin Testing System [Bio-Rad Laboratories, Hercules, CA, USA]. Cellulose acetate electrophoresis [pH 8.9] was performed, when required, to rule out the presence of abnormal hemoglobins. Genomic DNA was isolated from peripheral blood leucocytes using the QIAamp Blood Mini Kit [Qiagen GmbH, Hilden, Germany]. β-thalassemia mutations were characterized by reverse dot-blot hybridization^13^ or the amplification refractory mutation system [ARMS]^15^. Analysis of eight common deletional α thalassemia determinants [–α^3.7^, –α^4.2^, – –^SEA^, – –^THAI^, – –^FIL^, – –^MED^, – [α]^20.5^ and [– –^SA^] was performed using multiplex polymerase chain reaction^16^. The δ globin gene was sequenced on the ABI PRISM sequencer as described earlier [Applied Biosystems, Foster City, CA, USA]^17^.

### Statistical analysis

Statistical analysis of the data was performed using Graph Pad version 6.01 software (Graph Pad Prism Inc, California, U.S.A).

### Ethics approval

The study was approved by the National Institute of Immunohematology-Institutional Ethics Committee. All methods were performed in accordance with relevant guidelines and regulation.

### Consent to participate

Informed consent was obtained from all individual participants included in the study.

## Results

### HbA_2_ and MCV levels of the study subjects

**Figure 1:**
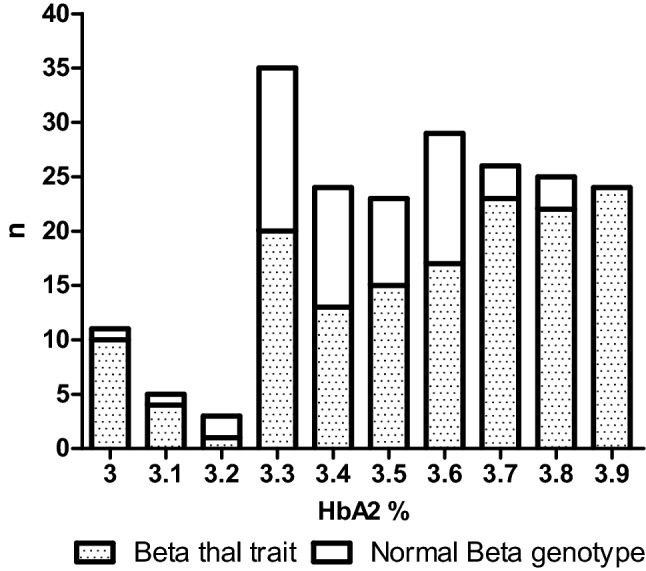
**Distribution of study population according to HbA**_**2**_** levels and β genotype**.

Of 205 individuals analysed in this study, 19 individuals had HbA_2_ ranging between 3.0 and 3.2%, 59 individuals had HbA_2_ ranging between 3.3 and 3.4% and 127 individuals had HbA_2_ ranging between 3.5 and 3.9%. Molecular analysis of the β globin gene revealed a β-thalassemia allele in 149 of 205 [73%] individuals and a normal β genotype in the remaining 56 [27%] individuals. Figure 1. shows the frequency of β-thalassemia carriers identified at different HbA_2_ levels investigated in our study.

### β-thalassemia mutations

Eleven β-thalassemia mutations were identified in the 149 carriers with borderline HbA_2_ levels (Table 1). β^+^ IVS1-5 G>C was the most common mutation (51%), followed by the milder β^++^ mutations such as Cap site +1 A>C, Poly A T>C and Poly A – AATAA, together accounting for 40% of carriers. β° mutations were identified in 9% of carriers with borderline HbA_2_ levels.

**Table 1:** Distribution of mutations among borderline HbA_2_ β-thalassemia carriers.

β-thalassemia mutation	n (%)	HbA_2_ 3.0–3.2%	HbA_2_ 3.3–3.4%	HbA_2_ 3.5–3.9%
IVS1-5 G > C [β^+]^	76 (51%)	3	18	55
Poly A T > C [β^++^ ]	26 (17.4%)	4	5	17
Cap site + 1 A > C[β^++ ]^	26 (17.4%)	6	6	14
Poly A—AATAA[β^++^]	8 (5.3%)	2	0	6
CD 15 G > A [β^0^]	6 (4%)	0	1	5
FS 16 –C [β^0^]	2 (1.3%)	0	1	1
CD 30 G > C [β^0^]	1 (0.6%)	0	1	0
CD 30 G > A [β^0^]	1 (0.6%)	0	0	1
CD 90 G > C [β^0^]	1 (0.6%)	0	0	1
FS 41/42CTTT [β^0^]	1 (0.6%)	0	1	0
FS 8/9 + G [β^0^]	1 (0.6%)	0	0	1
Total	149	15	33	101

In the HbA_2_ 3.0–3.2% range, 19 individuals were analysed and a β-thalassemia allele was identified in 15 individuals (79%). 12 of 15 β thalassemia heterozygotes showed presence of a β^++^ thalassemia allele [Cap + 1 A > C or Poly A T > C with co-inheritance of either − α^3.7^/αα deletion, HbA2 Saurashtra or IDA] and 3 of 15 β thalassemia heterozygotes showed presence of the β^+^ IVS1-5 G > C allele. β^0^ thalassemia defects were not identified in this group. Four individuals in this HbA_2_ group showed a normal β genotype of whom one was iron deficient and in the remaining three individuals, no defects could be identified.

In the HbA_2_ 3.3–3.4% range, 59 individuals were analysed and a β-thalassemia allele was identified in 33 individuals (56%). 11 of 33 β thalassemia heterozygotes showed presence of a β^++^ thalassemia allele [Cap + 1 A > C or Poly A T > C with either −α^3.7^/αα deletion or IDA], 18 of 33 β thalassemia heterozygotes showed presence of the β^+^ IVS1-5 G > C allele [with either − α^3.7^/αα deletion, HbA2 Yialousa, δ −68 C > T or IDA] and 4 of 33 β thalassemia heterozygotes showed presence of a β° thalassemia defect [FS 41/42 -CTTT, CD 30 G > C, CD 15 G > A with HbA2 Yialousa]. The remaining 26 of 59 individuals had a normal β genotype but 9 individuals showed presence of other defects [− α^3.7^/αα deletion, δ CD 20 T > A/HBD: c.62 T > A/HbA2 Roosevelt, δ promoter defect −68 C > T and novel δ CD 85 T > A defect]. No defects were identified in the remaining 17 of 26 individuals with a normal β genotype.

In the HbA_2_ 3.5–3.9% range, 127 individuals were analysed and a β-thalassemia allele was identified in 101 individuals (80%). 37 of 101 β-thalassemia heterozygotes showed presence of a β^++^ thalassemia allele (Cap + 1 A > C, Poly A T > C or Poly A –(AATAA) with presence of either −α^3.7^/αα deletion, −α^3.7^/−α^3.7^deletion, δ −68 C > T, HbA2 Saurashtra or IDA]. 55 of 101 β-thalassemia heterozygotes showed presence of a β^+^ thalassemia allele, IVS1-5 G > C [with presence of either α^3.7^/αα deletion, −α^3.7^/−α^3.7^ deletion, δ − 68 C > T, HbA2 Yialousa, novel δ globin gene defect CD 46 G > T/HBD: c.140G > T or IDA]. 9 β-thalassemia heterozygotes showed presence of a β^0^ mutation [CD 15 G > A, CD 30 G > A, FS 8/9 + G, FS 16 –C, CD 90 G > C with presence of either − α^3.7^/αα, δ − 68 C > T, δ CD 83 G > A/HBD:c.251G > A/ HbA2 Nishishinbashi or IDA]. The remaining 26 of 127 individuals showed a normal β genotype, but 9 individuals showed presence of other defects [α^3.7^/αα deletion or δ promoter defect −68 C > T].

**Figure 2:**
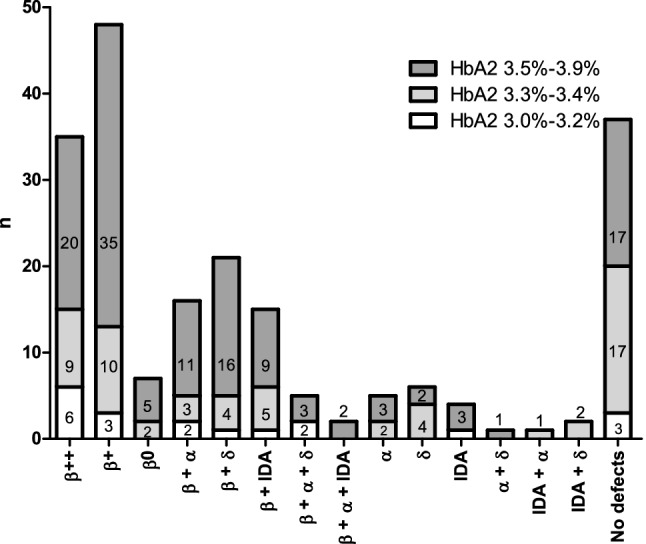
**Distribution of individuals based on HbA**_**2**_** levels and defects identified**. Number of study subjects showing different defects grouped according to their HbA_2_ levels. Blank boxes indicate n = 1.

Thus, overall, an acquired or genetic defect was identified in 168 of 205 (82%) individuals with borderline HbA_2_ levels. Among these 168 individuals, 19 were either iron deficient or showed α or δ globin gene defects and 149 (88%) were β-thalassemia carriers. 59 of 149 (40%) β-thalassemia heterozygotes showed co-existing IDA or co-inheritance of α or δ globin gene defects while 90 of 149 (60%) β-thalassemia heterozygotes did not have any co-existing defects. 101 of 149 β-thalassemia heterozygotes showed HbA_2_ 3.5–3.9% and 48 of 149 β-thalassemia heterozygotes showed HbA_2_ between 3.0 and 3.4%. Figure 2 shows the distribution of individuals investigated in this study.

### MCV-based analysis

Since MCV and MCH values, together with HbA_2_ levels, play a critical role in carrier diagnosis we analysed these parameters in the 149 β thalassemia heterozygotes identified in our study (Table 2). 10 of 15 β thalassemia heterozygotes with HbA_2_ between 3.0 and 3.2% showed MCV > 80 fL. Similarly, 10 of 33 β thalassemia heterozygotes with HbA_2_ between 3.3 and 3.4% showed MCV > 80 fL. Thus, 20 of 149 [14.0%] of all β thalassemia heterozygotes would be missed from being detected if MCV < 80 fL and HbA_2_ ≥ 3.5% were used as cut-off for carrier screening. These findings are of clinical significance because 5 of the 10 β thalassemia heterozygotes with HbA_2_ between 3.0–3.2% and MCV > 80 fL and 2 of the 10 β thalassemia heterozygotes with HbA_2_ between 3.3–3.4% and MCV > 80 fL were married to carriers of a hemoglobinopathy. A “missed” diagnosis of these 7 heterozygotes could have led to the birth of an affected child. Notably, of these 20 above mentioned β thalassemia heterozygotes with HbA_2_ between 3.0 and 3.4% and MCV > 80, 10 heterozygotes also showed MCH > 27 pg. These findings highlight the importance of performing a molecular work-up of the β globin gene in individuals showing borderline HbA_2_ levels especially if their partner is a known carrier of a hemoglobinopathy.

**Table 2:** Distribution of study individuals according to HbA_2_, MCV and MCH.

Parameters	HbA_2_ values (%)			Total
	3.0—3.2	3.3—3.4	3.5—3.9	
**Total individuals**	n = 19	n = 59	n = 127	n = 205
βTT				
MCV < 80 fL	5	23	87	115
MCV > 80 fL	10	10	14	34
Total βTT	15	33	101	149
MCH < 27 pg	10	21	91	122
MCH > 27 pg	5	12	10	27
Total βTT	15	33	101	149
**Normal β genotype**				
MCV < 80 fL	2	7	6	15
MCV > 80 fL	2	19	20	41
Total Normal β genotype	4	26	26	56
MCH < 27 pg	2	7	8	17
MCH > 27 pg	2	19	18	39
Total Normal β genotype	4	26	26	56

To understand the effect of the different genetic and acquired determinants on MCV levels of individuals with borderline HbA_2_ levels we then analysed our study population on the basis of MCV. Of the 205 individuals investigated in this study, 130 individuals showed MCV < 80fL and 75 individuals showed MCV > 80fL.

### MCV < 80 fL (n = 130)

A molecular defect could be identified in 123 of 130 individuals with borderline HbA_2_ levels and MCV < 80fL. Heterozygosity for β-thalassemia accounted for 93.4% (115 of 123) of defects identified. In this MCV group, the β^+^ IVS1-5 G > C mutation was identified in 60 of 115 (52%) carriers, β^++^ mutations were identified in 45 of 115 (39%) carriers and β^0^ mutations were identified in 10 of 115 (8.6%) carriers. Among the 115 β-thalassemia heterozygotes identified in this MCV group, 64 of 115 (56%) did not show presence of co-existing defects and 51 of 115 (44%) β-thalassemia heterozygotes showed concomitant IDA or co-inheritance of α or δ globin gene defects. A novel δ globin gene defect CD 46 G > T/HBD:c.140G > T was identified in a β-thalassemia heterozygote in this MCV group. In the eight individuals with a normal β genotype, α thalassemia was detected in two individuals, δ globin defects were identified in two individuals, IDA was noted in two individuals, one iron deficient sample showed the –α^4.2^/αα deletion and lastly one iron deficient sample showed the δ promoter defect −68 C > T. Genotypes and hematologic findings of these individuals are shown in Table 3.

**Table 3:** Hematological analysis of the individuals with MCV ≤ 80 fL and MCV ≥ 80 fL.

MCV ≤ 80 fL	n 130	RBC × 10^6^/µl		Hb g/dl		MCV fL		MCH pg	RDW %	HbA_2_ %	HbF %
No Defects identified	7										
Defects identified	123										
β^++^ thal	26	5.2 ± 0.6		11.87 ± 1.8		71.93 ± 4.77		23.04 ± 1.93	16.51 ± 4.84	3.5 ± 0.2	0.5 ± 1.00
β^+^ thal	34	5.1 ± 1.0		9.6 ± 1.8		64.4 ± 8.5		19.54 ± 3.55	20.86 ± 5.5	3.6 ± 0.3	1.3 ± 1.8
β^0^ thal	4	5.9 ± 0.9		11.25 ± 1.2		66.85 ± 4.00		19.92 ± 3.35	18.12 ± 4.55	3.5 ± 0.7	1.2 ± 2.1
β^++^ thal; α thal	7	5.54 ± 0.56		12.3 ± 1.24		70.8 ± 2.9		22.18 ± 3.2	15.5 ± 2.0	3.6 ± 0.1	0.4 ± 0.4
β^+^ thal; α thal	7	4.53 ± 0.92		8.86 ± 2.68		66.18 ± 9.7		19.63 ± 5.00	22.48 ± 5.75	3.5 ± 0.2	1.3 ± 0.7
β^++^ thal; δ thal	8	5.9 ± 0.5		12.61 ± 1.3		70.71 ± 3.64		21.18 ± 1.24	16.91 ± 2.15	3.6 ± 0.3	0.5 ± 0.3
β^+^ thal; δ thal	8	5.23 ± 1.61		10.9 ± 3.1		64.65 ± 16.5		19.9 ± 5.0	17.69 ± 5.7	3.4 ± 0.8	1.6 ± 2.0
β^0^ thal; δ thal	3	6.1 ± 0.2		11.7 ± 0.8		66.26 ± 6.9		19.2 ± 1.33	18.6 ± 1.3	3.5 ± 0.2	8.0 ± 10.94
β + thal; IDA	11	4.74 ± 0.4		7.9 ± 2.53		58.8 ± 9.5		16.63 ± 4.3	24.5 ± .4	3.5 ± 0.2	1.0 ± 0.5
β^++^ thal; α thal; δ thal	4	4.44 ± 0.35		9.8 ± 1.23		70.25 ± 6.7		22.2 ± 3.32	16.45 ± 2.7	3.4 ± 0.4	0.5 ± 0.4
β^0^ thal; α thal; δ thal	1	5.2		12.2		76.1		23.4	16.9	3.9	1.9
β° thal; δ thal; IDA	2	4.33 ± 1.3		8.6 ± 1.55		63.65 ± 6.5		31.75 ± 0.6	24.0 ± 3.8	3.6 ± 0.2	5.55 ± 5.72
α thal	2	5.2 ± 1.3		8.7 ± 5.94		62.26 ± 18.8		15.85 ± 7.4	20.65 ± 8.6	3.5 ± 0.2	0.8 ± 0.7
δ thal	2	5.9 ± 0.4		11.75 ± 1.4		63.25 ± 2.8		20 ± 1.27	16.25 ± 0.49	3.4 ± 0.1	4 ± 1.69
IDA	2	2.39 ± 0.4		5.7 ± 0.99		71.6 ± 3.2		23.85 ± 0.2	26.8 ± 9.7	3.4 ± 0.2	0.3 ± 0.4
IDA; α thal	1	2.23		5.8		79.8		26	27.4	3.3	0.3
IDA; δ thal	1	2.28		3.2		64.5		14	32.7	3.3	0

MCV ≥ 80 fL	n 75	RBC × 10^6^/µl	Hb g/dl		MCV fL		MCH pg		RDW %	HbA_**2**_ %	HbF %
No Defects identified	30										
Defects identified	45										
β^++^ thal	9	4.69 ± 0.6	13 ± 1.87		86.86 ± 4.7		27.8 ± 2.0		14.8 ± 2.7	3.1 ± 0.3	0.7 ± 0.4
β^+^ thal	14	4.18 ± 1.16	10.85 ± 2.7		88.6 ± 9.63		26.43 ± 3.83		20.13 ± 6.82	3.3 ± 0.2	0.75 ± 0.69
β^0^ thal	3	3.83 ± 0.5	11.2 ± 2.6		87 ± 9.44		31.1 ± 4.2		18 ± 6.47	3.7 ± 0.2	0.9 ± 1.4
β^++^ thal; α thal	2	4.05 ± 1.47	9 ± 1.2		83.55 ± 0.2		23.15 ± 5.3		24.25 ± 9.4	2.8 ± 0.8	1.3 ± 1.55
β^++^ thal; δ thal	1	5.41	14.3		83.5		26.4		13.6	3.6	0.0
β^+^ thal; δ thal	1	5.8	16		84.8		27.6		18.7	3.7	0.2
β^++^ thal; IDA	3	3.18 ± 1.77	7.33 ± 2.8		86.36 ± 4.6		24.6 ± 4.5		31.06 ± 10.5	3.2 ± 0.2	1.2 ± 1.2
β^+^ thal; IDA	1	1.23	3.7		120.4		39.8		33.0	3.6	0.0
α thal	3	2.62 ± 1.6	7.43 ± 4.49		91.96 ± 10.7		29.76 ± 3.98		17.9 ± 6.48	3.5 ± 0.1	0.6 ± 0.5
δ thal	4	3.91 ± 1.0	12.27 ± 3.5		92.47 ± 15.7		31.6 ± 6.85		15.1 ± 1.31	3.3 ± 0.5	0.5 ± 0.6
IDA	2	1.55 ± 0.5	4.95 ± 1.34		107.1 ± 10.1		32.45 ± 3.3		27.55 ± 2.75	3.5 ± 0.0	0.75 ± 0.3
α thal; δ thal	1	4.36	11.7		80.3		26.8		12.8	3.6	1.2
IDA; δ thal	1	2.4	7.3		100		32.9		38.9	3.3	0.7

### MCV > 80 fL (n = 75)

A molecular defect could be identified in 45 of 75 individuals with borderline HbA_2_ levels and MCV > 80fL. Heterozygosity for β-thalassemia accounted for 76% (34 of 45) of defects identified. In this MCV group, the β^+^ IVS1-5 G > C mutation was identified in 16 of 34 (47%) carriers, β^++^ mutations were identified in 15 of 34 (44%) carriers and β^0^ mutations were identified in 3 of 34 (8.7%) carriers. Among the 34 β-thalassemia heterozygotes identified in this MCV group, 26 (76%) did not show any co-existing defects and 8 of 34 (24%) β-thalassemia heterozygotes showed concomitant IDA, α or δ globin gene defects. In the remaining 11 individuals with a normal β genotype, three showed presence of α deletions and four showed a δ globin gene mutation, two individuals were iron deficient, one showed IDA; δ globin gene defect and one showed co-inheritance of α thalassemia and a δ globin gene defect. Genotypes and hematologic findings of these individuals are shown in Table 3.

**Figure 3:**
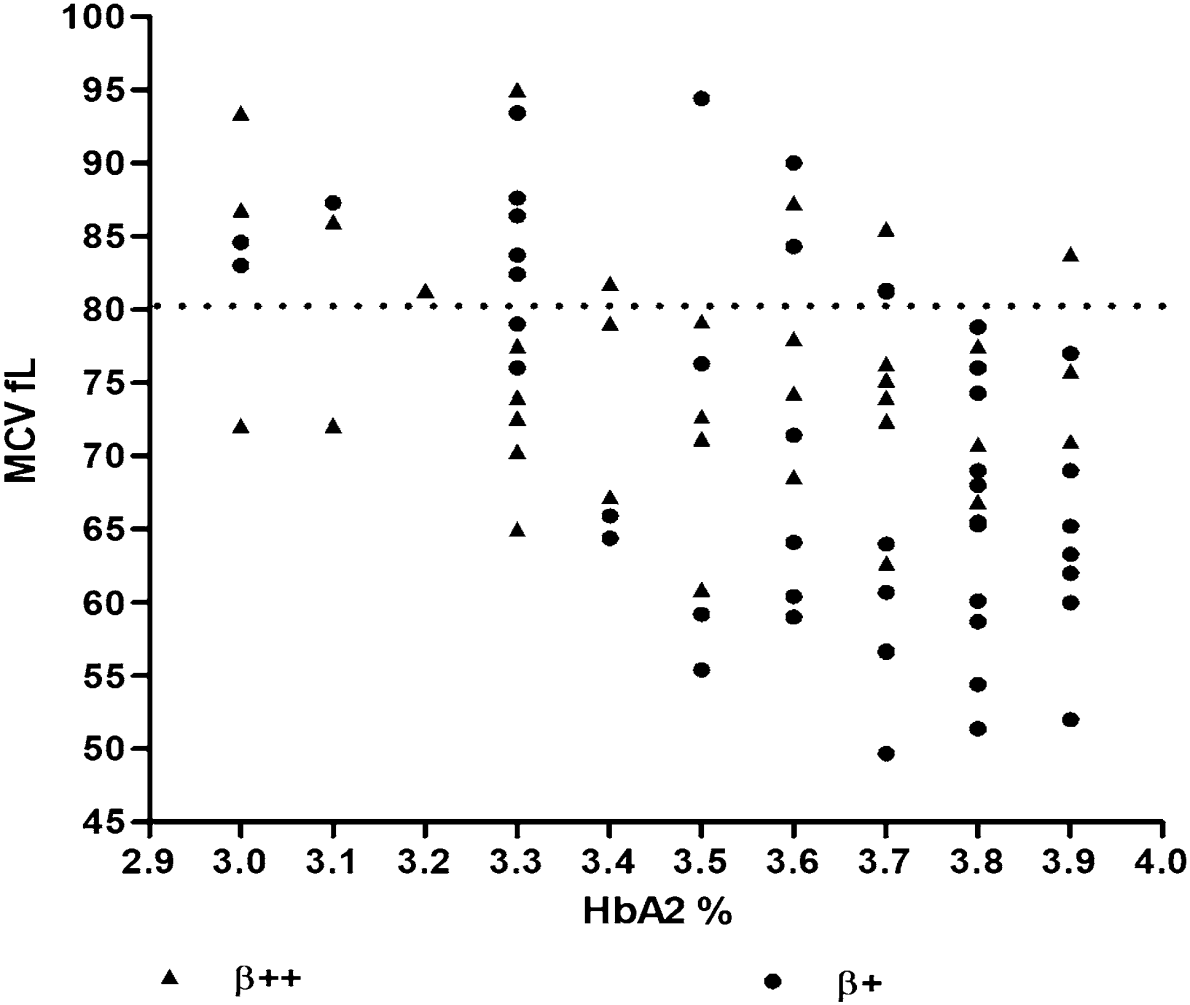
**Distribution of the MCV and HbA**_**2**_** levels of the two most common genotypes in borderline HbA**_**2**_** individuals with MCV < and > 80 fL.**

In our study, heterozygosity for β^+^ IVS1-5 G > C (52% and 47%) and heterozygosity for the β^++^ thalassemia mutations (47% and 44%) were the most prevalent defects in individuals with MCV < 80fL and MCV > 80fL, respectively. To get a better understanding of the distribution of MCV and HbA_2_ levels of these heterozygotes we plotted a scatter diagram of the MCV and HbA_2_ levels of the carriers (Fig. 3). From the plot we observe that carriers of β^+^ IVS1-5 G > C and β^++^ defects show largely similar HbA_2_ levels and MCV values and note the absence of IVS1-5 G > C β thalassemia carriers with MCV < 80 and HbA_2_ < 3.3%.

To summarize our findings, we show that individuals with borderline HbA_2_ levels are not rare in the Indian population with approximately 82% [168/205] of them harbouring a molecular or acquired defect. We found that at all HbA_2_ levels investigated in this study, and irrespective of MCV (< 80 or > 80 fL), heterozygosity for β-thalassemia was the most common defect. The severe β^+^ thalassemia allele, IVS1-5 G > C, was identified in heterozygotes with MCV < 80 fL and HbA_2_ > 3.5% as well as in heterozygotes with MCV > 80 fL and HbA_2_ < 3.5%. We found that the co-inheritance of α or δ globin gene defects was more common in individuals with borderline HbA_2_ levels and MCV < 80fL (12.1% and 16.5%, respectively) as compared to that in individuals with borderline HbA_2_ levels and MCV > 80fL (5.9% and 5.9%, respectively). 40% of individuals with borderline HbA_2_ levels and MCV > 80fL did not show any defect. Since most of the defects identified were common to all three HbA_2_ groups and both MCV groups we were unable to associate any defect exclusively to a particular HbA_2_ or MCV group. In the course of our study we identified 48 β-thalassemia heterozygotes who showed HbA_2_ 3.0–3.4%. Of these 48 heterozygotes, 20 also showed MCV > 80fL. These cases emphasize that a molecular work up of the β globin gene is the only way to achieve a confident diagnosis of individuals with borderline HbA_2_ levels.

## Discussion

Individuals affected by β-thalassemia major require regular blood transfusions and lifelong medical care to survive. Research in ameliorating the pathological effects and in the treatment of the disease are ongoing^18^, however, the newer treatment modalities are high-priced and often unaffordable to the general population in low and middle income countries. It cannot be stressed enough that this complex disease is preventable if simple and cost-effective measures such as carrier screening and genetic counselling are rigorously employed.

The heterozygous form of β-thalassemia is associated with a mild persistent anemia and distinctly elevated levels of HbA_2_, which form the basis for screening programs world-over. Approximately 80–90 million people worldwide are carriers of β-thalassemia^19^ with India alone harbouring 35–45 million carriers^14^. From this perspective, the implications of borderline HbA_2_ levels in the diagnosis of β-thalassemia holds immense significance. In this study we have identified the factors responsible for borderline HbA_2_ levels, have investigated the effects of confounding factors on HbA_2_ and MCV levels and have interpreted the effects of these parameters in β-thalassemia carrier screening.

Heterozygosity for β-thalassemia was the most common cause of borderline HbA_2_ levels in our study population. 40% β-thalassemia heterozygotes showed concomitant presence of IDA or α or δ globin gene defects while 60% heterozygotes showed absence of these confounding factors.

Presently, our lab and different laboratories worldwide consider HbA_2_ > 3.2% as the second level of diagnosis of β thalassemia. However, during the time this study was conducted (2009–2013) our laboratory and other laboratories offering β thalassemia screening^4,21^ used to consider HbA_2_ ≥ 4.0% as cut-off for diagnosis of heterozygous β thalassemia which is why, in this study, we have considered borderline HbA_2_ levels as 3.0–3.9%.

On stratifying the β-thalassemia heterozygotes on the basis of HbA_2_, we found that 32% had HbA_2_ levels in the range of 3.0–3.4% and 68% had HbA_2_ levels 3.5–3.9%. Several laboratories consider HbA_2_ ≥ 3.5% as the cut-off for identification of β-thalassemia carriers during screening programmes and in the absence of molecular testing, had HbA_2_ levels been the sole diagnostic determinant for β-thalassemia carriers, the 48 heterozygotes showing HbA_2_ 3.0–3.4% would be misdiagnosed as non-carriers [false negatives]. Interestingly, 15 of the 48 false negative heterozygotes were partners of HbS or β-thalassemia heterozygotes. 20 of these 48 false negative heterozygotes showed MCV > 80fL and 10 of these 20 individuals also showed MCH > 27 pg. These findings highlight how, despite using a combination of MCV and HbA_2_ values (and sometimes, even MCH) for screening, β thalassemia heterozygotes could still be misdiagnosed as non-carriers. As documented by Giambona et al.^1^, it is important and highly relevant to detect all β-thalassemia carriers for a prevention screening program aimed at the identification of at-risk couples. Collectively, our findings emphasize the need to offer molecular screening of the β globin gene to partners of carriers of hemoglobinopathies, irrespective of their hematological indices and HbA_2_ levels, to avoid a misdiagnosis of these *“at-risk”* couples who, in turn, could have affected children. Our findings are supported by a study by Gorivale et al., who reported that 3.4% couples referred for β-thalassemia screening had one partner with a normal/borderline HbA_2_ level [1.0% – 3.5%] in a routine diagnostic laboratory setting^20^. Gorivale et al. identified 73% partners with a normal/borderline HbA_2_ level as β-thalassemia heterozygotes [Cap + 1 A > C—60%, IVS1-5 G > C—17%, Poly A T > C—8%, and CD 15 G > A, CD 16 -C and CD 30 G > C mutations each at 4%]. Another study by Nadkarni et al. 2019 showed HbA_2_ levels between 1.0–3.9% in 131 β-thalassemia heterozygotes with variable MCV and MCH levels heterozygous for eight different β-thalassemia mutations^13^.

In our study, 11 β-thalassemia mutations were associated with borderline HbA_2_ levels. Five mutations identified in our borderline HbA_2_ individuals overlapped with the spectrum of common β-thalassemia mutations in India (IVS1-5 G > C, IVS1-1 G > T, CD 8/9 + G, CD 41/42 -CTTT, CD 15 G > A and CD 30 G > C). We identified β^++^, β^+^ and β^0^ thalassemia mutations in our borderline HbA_2_ β-thalassemia heterozygotes. A study by Rangan et al.^12^ has previously also identified β^++^, β^+^ or β^0^ thalassemia defects in 8 of 25 [32%] individuals with HbA_2_ 3.0 – 4.0%. Association of β^0^ and β^+^ thalassemia mutations in borderline HbA_2_ individuals is also not uncommon in other populations^21–23^. Overall, β^+^ IVS1-5 G > C mutation was the most common defect identified in borderline HbA_2_ individuals in our study [51%], followed by the milder β^++^ thalassemia mutations [40%] and the β^0^ thalassemia mutations [9%].

We found that at lower HbA_2_ levels (3.0–3.2%), the β^++^ thalassemia alleles are more common, β^0^ thalassemia alleles did not occur at HbA_2_ < 3.2% but as HbA_2_ levels increase (3.3-3.9%) the number of heterozygotes for β^+^ IVS1-5 G > C increase. These findings can be explained by the fact that IVS1-5 G > C mutation is the most common β-thalassemia mutation in the Indian population with a prevalence of about 60% in Western India^24^. Our study for the first time highlights that this severe β^+^ thalassemia allele that has been conventionally associated with elevated HbA_2_ levels in past studies may also be the most common allele in borderline HbA_2_ carriers in India. Indeed, heterozygosity for a β^+^ thalassemia allele, IVS1-6 T > C, is reported as the most common cause of borderline HbA_2_ levels in the Mediterranean region^1,25^.

Many factors influence HbA_2_ levels besides the β-thalassemia alleles, such as α-thalassemia, δ-thalassemia and severe IDA^26^. The co-inheritance of α- or δ- thalassemia in β-thalassemia carriers has also been reported to lower/normalize HbA_2_ levels^25,27,28^. To identify if these genotypes were associated with HbA_2_ levels in our study population, we analysed their α and δ globin genotypes and evaluated the presence of iron deficiency anemia.

A high prevalence of associated α thalassemia is reported in Indian β-thalassemia carriers^29^. In our study, the co-inheritance of α thalassemia was noted in 10.7% β-thalassemia heterozygotes. A recent study from Thailand has reported that as many as 43.75% β thalassemia heterozygotes with HbA_2_ 3.5–3.9% also co-inherited α thalassemia^30^. Reports also show that co-inheritance of α- and β-thalassemia could normalize MCV and MCH levels leading to misdiagnosis^28,31^. We did not observe such an effect in our study: of the 16 α and β-thalassemia double heterozygotes identified in our study, 14 showed MCV < 80 fL and 14 showed MCH < 27 pg. Another group has also reported a significant improvement in MCV and MCH values due to β^+^ or β° thalassemia mutations interacting with one or two α globin gene abnormalities [(− α/αα), (α^T^α/αα) or (− − /αα)] in the Thai population^32^. In our study, of the seven β^+^ thalassemia heterozygotes with co-inherited α thalassemia, five showed MCV 61.5 ± 4.6 fL and MCH 17.62 ± 2.25 pg and two showed MCV 78.0 ± 0.0 fL and MCH 25.05 ± 5.16 pg. One β° thalassemia heterozygote with co-inherited α thalassemia showed MCV 76.1 fL and MCH 23.4 pg. Overall, it appears that the co-inheritance of α thalassemia did not normalize MCV or MCH values in borderline HbA_2_ β-thalassemia carriers in our study.

The co-existence of δ thalassemia in *cis* or in *trans* leading to reduction of HbA_2_ levels and a change in typical hematological phenotype of β-thalassemia trait is not uncommon^1,25,27,33^. Co-inheritance of δ thalassemia was noted in 14% β-thalassemia heterozygotes in our study. The defects identified included δ promoter defects −68 C/T and −68 T/T, HbA2 Yialousa, CD 83 G > A/HBD:c.251G > A/ Hb A2 Nishishinbashi, a novel δ globin gene defect CD 46 G > T/HBD: c.140G > T, and a δ globin structural variant; HbA2 Saurashtra^34^. MCV of the β and δ double heterozygotes was lower (Mean ± SD, 69 ± 7.9fL) than that of β-thalassemia carriers without any other defect (Mean ± SD, 73.53 ± 11.8fL) although the difference was not statistically significant (p = 0.13).

India is one of the countries with the highest prevalence of β-thalassemia and anemia and hence it is not uncommon to identify iron deficient β-thalassemia carriers. IDA is associated with a melange of red cell abnormalities coupled with decreases in HbA_2_ that may at times lead to misdiagnosis of β-thalassemia carriers^35^. In borderline HbA_2_ β thalassemia carriers, the significant iron depletion caused by IDA reduces the already poorly elevated HbA_2_ fraction of hemoglobin into the normal range^36^. However, other studies dispute the decrease in HbA_2_ levels in the presence of iron deficiency^37,38^. In our study, co-existence of IDA was noted in 10% β-thalassemia heterozygotes with HbA_2_ between 3.0 and 3.9%. HbA_2_ levels of β thalassemia heterozygotes with co-existing IDA was not different (Mean ± SD, 3.5 ± 0.2%) from that of β-thalassemia carriers without any other defect (Mean ± SD, 3.5 ± 0.2%). However, we noticed that the mean HbA_2_ values of the iron deficient β^++^ thalassemia heterozygotes were 3.2 ± 0.2% and that of iron deficient β^+^ thalassemia heterozygotes at 3.6 ± 0.2% (p = 0.0459). The co-existence of IDA in β^++^ thalassemia heterozygotes appears to decrease HbA_2_ levels in our study population.

On assessing our study population on the basis of MCV, we found that 93% individuals with MCV < 80 fL and 76% individuals with MCV > 80 fL were β-thalassemia carriers. In both MCV groups, heterozygosity for β^+^ IVS1-5 G > C and for the β^++^ mutations were the most common etiologies associated with borderline HbA_2_ levels. A previous study^1^ has suggested that with the evaluation of both MCV and HbA_2_, it is possible to differentiate mild mutations from more severe β globin gene defects. Notably, in this study^1^ the β^0^ and β^+^ alleles were associated with MCV < 80fL while the β^++^ alleles were noted in both MCV groups. In our study, heterozygotes for all three types of β thalassaemia alleles (β^0^, β^+^ and β^++^ ) could be identified in both MCV groups. We also identified a subset of β^+^ and β^++^ thalassemia heterozygotes showing MCV > 80fL with HbA_2_ levels < 3.5% (Fig. 3.) in our study population. These atypical carriers can be missed if molecular analyses of the β globin gene is not undertaken in a laboratory setting and may potentially add to the β thalassemic burden of the nation. In our study, associated α thalassemia [MCV < 80fL group 12% versus MCV > 80fL group 6%] and δ thalassemia [MCV < 80fL group 16% versus MCV > 80fL group 6%], were more prevalent in β thalassemia heterozygotes showing MCV < 80 fL and MCH < 27 pg. Our findings suggest that both co-inheritance of α and δ in borderline HbA_2_ β thalassemia heterozygotes reduce MCV and MCH.

In this study, we also identified 26 of 205 individuals showing HbA_2_ between 3.5 and 3.9% with a normal β genotype, of whom six individuals had MCV < 80 fL (false positives). Three of these six false positive individuals showed presence of IDA, α thalassemia and δ thalassemia while no defects could be identified in the remaining three individuals. Another Indian study^11^ also reported borderline HbA_2_ levels [3.5–3.9%] in 3.6% individuals with absence of a β-thalassemia defect. The probable etiologies in such individuals have been discussed by Giambona et al.^1^. Individuals with MCV < 80 fL are thought to harbour rare globin gene defects, sequence changes in locus control regions or enhancer regions of the β globin gene while individuals with MCV > 80 fL are speculated to be the result of increased δ globin gene expression, use of antiretroviral drugs, presence of co-morbidities such as hyperthyroidism or genetic disorders such as Pseudoxanthoma Elasticum or defects in genes regulating synthesis of specific protein factors.

Few studies, so far, have delineated the molecular basis of borderline HbA_2_ levels in different populations as discussed in a recent review^10^. Results from these studies collectively reveal that the causes of borderline HbA_2_ levels may include heterozygosity for β-thalassemia, presence of KLF1 gene mutations, α thalassemia, co-inheritance of β and δ thalassemia, co-inheritance of β and α thalassemia, α globin gene triplication or the presence of hereditary persistence of fetal hemoglobin. In our study, the most common causes of borderline HbA_2_ levels were heterozygosity for β-thalassemia, co-inheritance of β and δ- thalassemia, co-inheritance of β- and α-thalassemia and the co-existence of IDA in β-thalassemia carriers. A recent study by Hariharan et al.^39^ in the Indian population has demonstrated the prevalence of KLF1 gene variations to be 7.6% in individuals with borderline HbA_2_ levels [3.3–3.9%]. Although a limitation of the present study remains the non-assessment of KLF1 gene mutations and α gene triplications, we have for the first time delineated the molecular basis of normal/borderline HbA_2_ levels in the Indian population. We have described the potential of missing β-thalassemia carriers when HbA_2_ levels alone or in combination with MCV are used as stand-alone diagnostic determinants. We strongly recommend a comprehensive molecular work up of the β globin gene in individuals with borderline HbA_2_ levels with special attention to cases with a hemoglobinopathy carrier partner.

## Conclusions

Borderline HbA_2_ levels are one of the cause of misdiagnosis of β-thalassemia carriers. Our study puts forth preliminary data on the spectrum of defects associated with borderline HbA_2_ levels. Heterozygosity for β-thalassemia remains the single largest cause of borderline HbA_2_ levels in our study, however, the interaction of α, δ or IDA in β-thalassemia heterozygotes as a cause of borderline HbA_2_ levels is not uncommon. We identified cases where HbA_2_ levels, MCV and MCH levels failed to predict heterozygosity for β-thalassemia and thus could lead to misdiagnosis of carriers. We strongly suggest that a comprehensive molecular work up of the β globin gene should be performed when borderline HbA_2_ levels, in combination with normal/subnormal MCV and MCH values, are encountered in the routine laboratory setting in cases where one partner is a carrier of a hemoglobinopathy. This will not only reduce the false negative results, but also reduce the burden of thalassemia in the future.
